# Treatment Switching and Discontinuation Over 20 Years in the Big Multiple Sclerosis Data Network

**DOI:** 10.3389/fneur.2021.647811

**Published:** 2021-03-17

**Authors:** Jan Hillert, Melinda Magyari, Per Soelberg Sørensen, Helmut Butzkueven, Anneke Van Der Welt, Sandra Vukusic, Maria Trojano, Pietro Iaffaldano, Fabio Pellegrini, Robert Hyde, Leszek Stawiarz, Ali Manouchehrinia, Tim Spelman

**Affiliations:** ^1^Department of Clinical Neuroscience, Karolinska Institute, Stockholm, Sweden; ^2^The Danish Multiple Sclerosis Registry, Department of Neurology, Rigshospitalet, Copenhagen, Denmark; ^3^Department of Neurology, Danish Multiple Sclerosis Center, Rigshospitalet, University of Copenhagen, Copenhagen, Denmark; ^4^MSBase Foundation, Melbourne, VIC, Australia; ^5^Multiple Sclerosis and Neuroimmunology Research, Central Clinical School, Alfred and Box Hill Hospitals, Monash University, Melbourne, VIC, Australia; ^6^Service de Neurologie, sclérose en plaques, pathologies de la myéline et neuro-inflammation, and Observatoire Français de la Sclérose en Plaques, Hôpital Neurologique Pierre Wertheimer, Hospices Civils de Lyon, Lyon, France; ^7^Centre des Neurosciences de Lyon, INSERM 1028 et CNRS UMR5292, Lyon, France; ^8^Université Claude Bernard Lyon 1, Faculté de Médicine Lyon-Est, Lyon, France; ^9^Department of Basic Medical Sciences, Neurosciences, and Sense Organs, University of Bari Aldo Moro, Bari, Italy; ^10^Biogen International GmbH, Zug, Switzerland

**Keywords:** multiple sclerosis, disease modifying treatment, big MS data, registry study, treatment interruption and discontinuation

## Abstract

**Background:** Although over a dozen disease modifying treatments (DMTs) are available for relapsing forms of multiple sclerosis (MS), treatment interruption, switching and discontinuation are common challenges. The objective of this study was to describe treatment interruption and discontinuation in the Big MS data network.

**Methods:** We merged information on 269,822 treatment episodes in 110,326 patients from 1997 to 2016 from five clinical registries in this cohort study. Treatment stop was defined as a clinician recorded DMT end for any reason and included treatment interruptions, switching to alternate DMTs and long-term or permanent discontinuations.

**Results:** The incidence of DMT stopping cross the full observation period was lowest in FTY (19.7 per 100 person-years (PY) of treatment; 95% CI 19.2–20.1), followed by NAT (22.6/100 PY; 95% CI 22.2–23.0), IFNβ (23.3/100 PY; 95% CI 23.2–23.5). Of the 184,013 observed DMT stops, 159,309 (86.6%) switched to an alternate DMT within 6 months. Reasons for stopping a drug were stable during the observation period with lack of efficacy being the most common reason followed by lack of tolerance and side effects. The proportion of patients continuing on most DMTs were similarly stable until 2014 and 2015 when drop from 83 to 75% was noted.

**Conclusions:** DMT stopping reasons and rates were mostly stable over time with a slight increase in recent years, with the availability of more DMTs. The overall results suggest that discontinuation of MS DMTs is mostly due to DMT properties and to a lesser extent to risk management and a competitive market.

## Introduction

Multiple sclerosis (MS) is a life-long disease where disability typically develops over decades. For over 20 years, disease modifying treatments (DMTs) have been available to reduce attack frequencies, focal inflammatory brain lesions and development of disability. Although over a dozen DMTs are available for relapsing forms of multiple sclerosis (MS), discontinuation of treatment is a common challenge as consistent control of inflammation is a priority (1–5). Conversely, the broader availability of an increasingly diverse range of treatment options provides opportunities for better management in patients able to switch. Changes in product availability, reimbursement and treatment recommendations have led to a growing interest for identifying reliable predictors of DMT discontinuation (6–9). Furthermore, discontinuation on a prescribed drug may differ between specific drugs, between countries, over time and calendar year (of licensing new drug) (10–12).

The growth of real-world clinical MS databases, particularly large, longitudinal disease registries, provide a unique opportunity to describe discontinuation trends over large patient numbers and long-term observation periods (13, 14). The objective of this study was to describe the frequency of DMT discontinuation recorded across the pooled Big MS Data Network (BMSD) and to descriptively compare patterns of discontinuation between different time periods and treatment epochs.

## Materials and Methods

### Data

The project was conducted using data from the five clinical MS registries included in the BMSD network project: the Italian MS registry, OFSEP of France, the Danish and Swedish national MS registries and the international MSBase database. Treatment episodes and associated patient data complying to minimum dataset requirements were individually extracted from the five contributing datasets and then pooled into a single combined dataset. Minimum dataset requirements included treatment information (product, treatment start and end dates and reason for treatment interruption/discontinuation), demography (age and sex) and clinical and disease characteristics (date of first symptoms, date of MS diagnosis, EDSS).

### Quality Checks

Data quality checks were conducted prior to merging to minimize outcome assessment and follow-up bias in the cohorts under study. This included ensuring all variables required for the minimum analysis dataset had been extracted and transferred in the correct format, consistent across all five registries. The data was then checked for duplicates and date inconsistencies covering key demographic, disease and treatment dates. Data counts were then performed to assess completeness of key variables.

### Patients and Observation Period

Diagnosis of MS was confirmed according to Poser or McDonald criteria. All eligible MS patients for whom at least one DMT had been initiated during the relapsing remitting MS stage of disease were included in the analysis. Subjects were considered exposed to a DMT if they had received at least one injection/infusion (or at least a one-time consumption of an oral drug). We defined a pre-study period preceding the index date during which patients were required to have continuous medical service coverage. This was defined as a minimum of 6 months. The pre-study period ensured a standard run-in period without DMT exposure and a standard period during which the diagnosis of MS was identified. Patients not diagnosed as RRMS or RRMS patients not initiating a DMT therapy during the RRMS phase were excluded from the analysis. The observation period of the study was January 1st 1996 through 31st December 2016. Patients were included in the analysis if they recorded at least one qualifying DMT episode at any stage during the observation period and were not required to be active registry follow-up for the entirety of the observation period. A subset of patients from the Italian registry are also tracked in MSBase. To avoid duplicate patient records these patients were removed from the MSBase contribution to the overall pooled sample.

### Definitions

A treatment episode was defined as the time from clinician recorded DMT start to clinician recorded DMT stop. For convenience, we have referred here to all DMT interruptions, switching and discontinuations as treatment changes. Treatment stop was defined as a clinician recorded DMT end for any reason. This included both DMT stops that were followed by a switch or change to an alternate DMT and DMT stops that were followed by no treatment for the remainder of a patient's follow-up. A treatment switch was defined as a gap of no >6 months between ceasing a DMT and initiating an alternate DMT. Changes of dose of an existing DMT or the addition of a second drug were not considered to represent discontinuations. DMTs included in the analysis were categorized as followed: IFNβ-1a IM, IFNβ-1a SC, IFNβ-1b, glatiramer acetate, IFNβ-1a not further specified, natalizumab, rituximab, fingolimod, dimethyl fumarate, teriflunomide, alemtuzumab and other. Reasons for discontinuation were analyzed as reported by the contributing registries.

### Ethics Statement

The datasets presented in this article are not readily available secondary to the governing rules of the contributing registries that prohibit the sharing of both patient level data and aggregate data that may identify individual patients. Selected data and analyses may be accessed *via* direct request to the individual registries and subject to satisfying the data sharing permission rules of each registry.

### Statistical Analyses

This analysis was descriptive only. Categorical variables were summarized using frequency and percentage. Continuous variables were summarized using mean and standard deviation (SD) or median and inter-quartile range (IQR) as appropriate. All analyses were conducted in R (R Foundation for Statistical Computing).

## Results

A total of 110,326 patients contributing 269,822 DMT treatment episodes from the five registries were included in the analysis (Table 1). Across the pooled sample, females accounted for 78,269 (70.9%) of included patients. This proportion was similar across cohorts ranging from a low of 67.9% in the Italian cohort to a high of 74.5% in the French OFSEP cohort. Mean (SD) age at MS onset was 30.9 years (10.3). Mean (SD) age at DMT initiation was 36.6 years (11.0) across the entire cohort. Across the observation period, Scandinavian patients tended to be treated later, with Danish patients initiating DMT at a mean (SD) of 38.3 (12.8) years of age and Swedish patients starting treatment at a mean (SD) of 40.7 (12.4) years. This compares to a mean of 35.5, 35.8 and 36.3 years, respectively, for the MSBase, Italian and French cohorts, respectively. Across the full observation period, mean (SD) treatment duration was comparable across all five registries, ranging from 2.06 (2.12) years in Italian patients up to 2.82 years (2.38) in the Danish cohort.

**Table 1:** Treatment episodes and discontinuations by registry.

	**Category**	**Denmark**	**Sweden**	**OFSEP**	**Italy**	**MSBase**	**Total**
PATIENT CHARACTERISTICS^*^							
Patient count – n		7990	15983	24616	26985	34752	110326
Sex – n (%)	Female	5485 (68.7)	11245 (70.4)	18333 (74.5)	18315 (67.9)	24891 (71.6)	78269 (70.9)
	Male	2505 (31.4)	4738 (29.6)	6283 (25.5)	8670 (32.1)	9861 (28.4)	32057 (29.1)
Age at MS onset (years) – mean (SD)		32.8 (9.9)	34.4 (12.8)	31.1 (9.5)	29.6 (9.7)	30.5 (9.9)	30.9 (10.3)
Age at first DMT (years) – mean (SD)		38.3 (12.8)	40.7 (12.4)	36.3 (10.3)	35.8 (10.7)	35.5 (10.7)	36.6 (11.0)
**TREATMENT CHARACTERISTICS**							
Treatment episodes – n		14252	38229	65535	79816	71990	269822
Discontinuations – n (%)		8936 (62.7)	24704 (64.6)	45966 (70.1)	59590 (74.7)	44817 (62.3)	184013 (68.2)
Treatment duration (years) – mean (SD)		2.82 (2.38)	2.31 (2.24)	2.18 (2.18)	2.06 (2.12)	2.29 (2.20)	2.23 (2.20)

* *Count of individual patients contributing at least 1 treatment episode to the analysis*.

A total of 184,013 (68.2%) DMTs were stopped during the observation period. IFNβ accounted for the largest proportion of observed treatments in the pooled data (116,551 treatment episodes; 43.2%), followed by natalizumab (NTZ) (33,974; 12.6%), glatiramer acetate (GLA) (32,324; 12.0) and fingolimod (FTY) (19,675; 7.3%). The incidence of DMT stopping across the full observation period was lowest in FTY (19.7 stops per 100 person-years (PY) of treatment; 95% CI 19.2–20.1), followed by NAT (22.6/100 PY; 95% CI 22.2–23.0), IFNβ (23.3/100 PY; 95% CI 23.2–23.5) and GLA (25.8/100 PY; 95% CI 25.4–26.2). Across the pooled data there was a wide variety of treatment pathways taken by patients following stopping of first line therapy. Of the 184,013 observed DMT stops, 159,309 (86.6%) switched to an alternate DMT within 6 months of discontinuation. The most frequent switch products across the observation period were an alternate platform (IFNβ or glatiramer), natalizumab or fingolimod.

**Figure 1:**
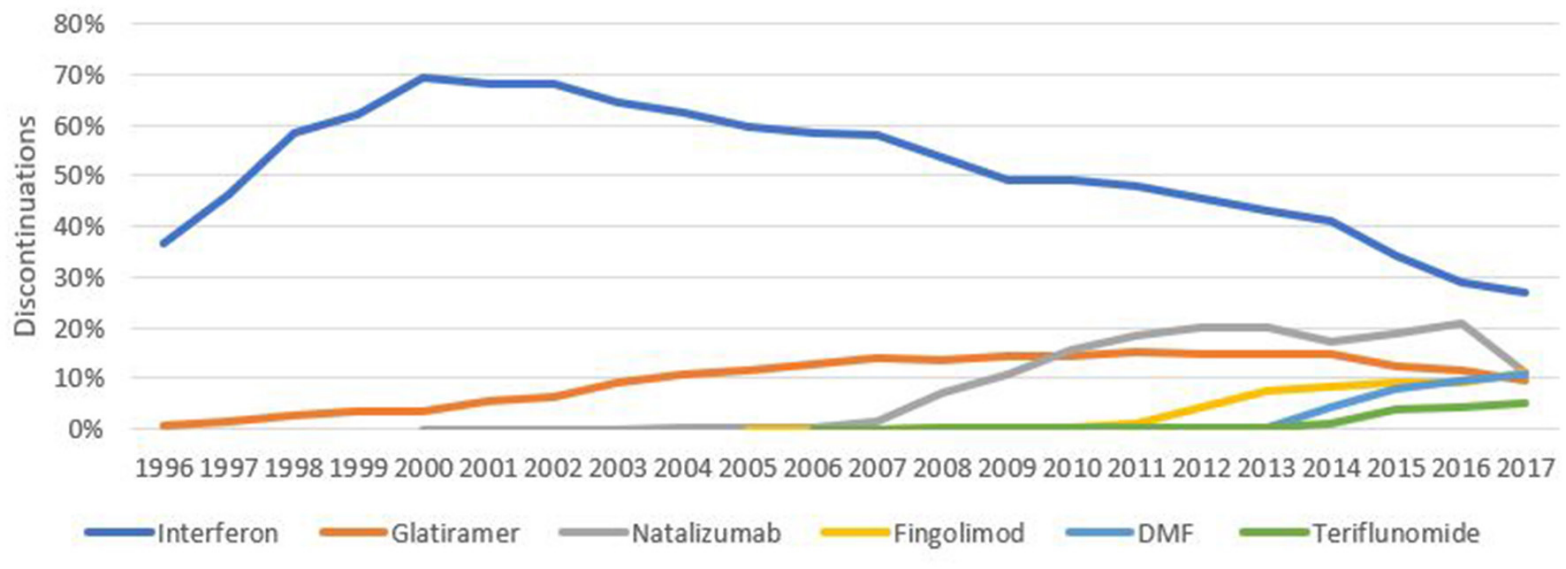
Discontinuations per product per year as a proportion of all annual discontinuations.

Consistent with being the most frequently prescribed DMT, IFN was the most frequently discontinued DMT in each of the years between 1996 and 2017 inclusive (Figure 1), accounting at its peak for 69.1% (2,230/3,228) of all DMT stops that occurred during the year 2000. IFN accounted for the majority of the market share at this time, accounting for 76.7% (4,743/6,181) of all patients treated with DMT at the beginning of 2000. This gradually fell to a low of 26.8% of all treatment interruptions (922/3,441) in 2017. Glatiramer acetate was the second most frequently discontinued DMT between 1996 and 2009 (accounting for 7,287/69,647 treatment interruptions during this period), before being overtaken by natalizumab in 2010, which contributed 19% (2,026/10,680) of DMT stops during that year. By 2017, four drugs (NTZ 11.1%, FTY 11.1%, Dimethyl Fumarate 10.8% and GLA 9.4%) accounted for almost equal proportions of DMT stops, accounting for 319, 319, 310, and 270 of the total 2,870 treatment interruptions recorded in that year. Of all patients on DMT at the start of 2017, FTY, GLA, NTZ, and DMF accounted for 16.0, 12.3, 9.1, and 6.2% of these treatments, respectively.

### Persistence

**Figure 2:**
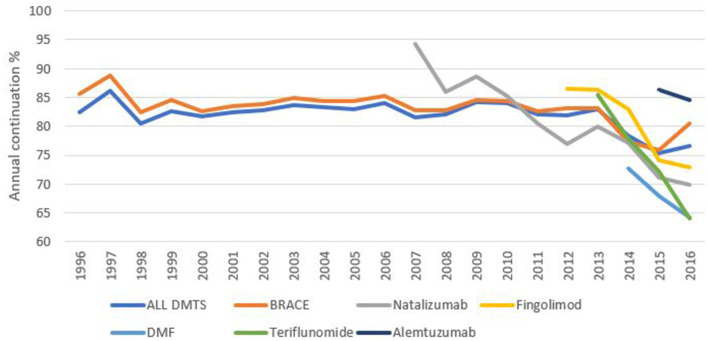
Annual continuation percentages by DMT. Defined as the number of patients persisting on DMT for a full calendar year divided by the total number of patients on that DMT as at January for any given year.

A key goal in MS management is to provide patients with a DMT they are likely to continue on over a longer period of time. Figure 2 illustrates the treatment-specific chance of continuing for another year on (a) all drugs combined as well as for (b) platforms (IFNβ-1a IM, IFNβ-1a SC, IFNβ-1b, and glatiramer acetate) or (c) natalizumab. Interestingly, overall continuation is fairly stable until the last few years when a decrease was observed in 2014 and 2015 for all drugs combined as well as for the platform DMT group. In contrast, the annual chance of continuing on natalizumab has gradually decreased from 95% during its first year to 70% in 2016, likely as a consequence of a gradual implementation of a risk management scheme to prevent progressive multifocal leukoencephalopathy (PML).

### Reason for DMT Stopping

**Figure 3:**
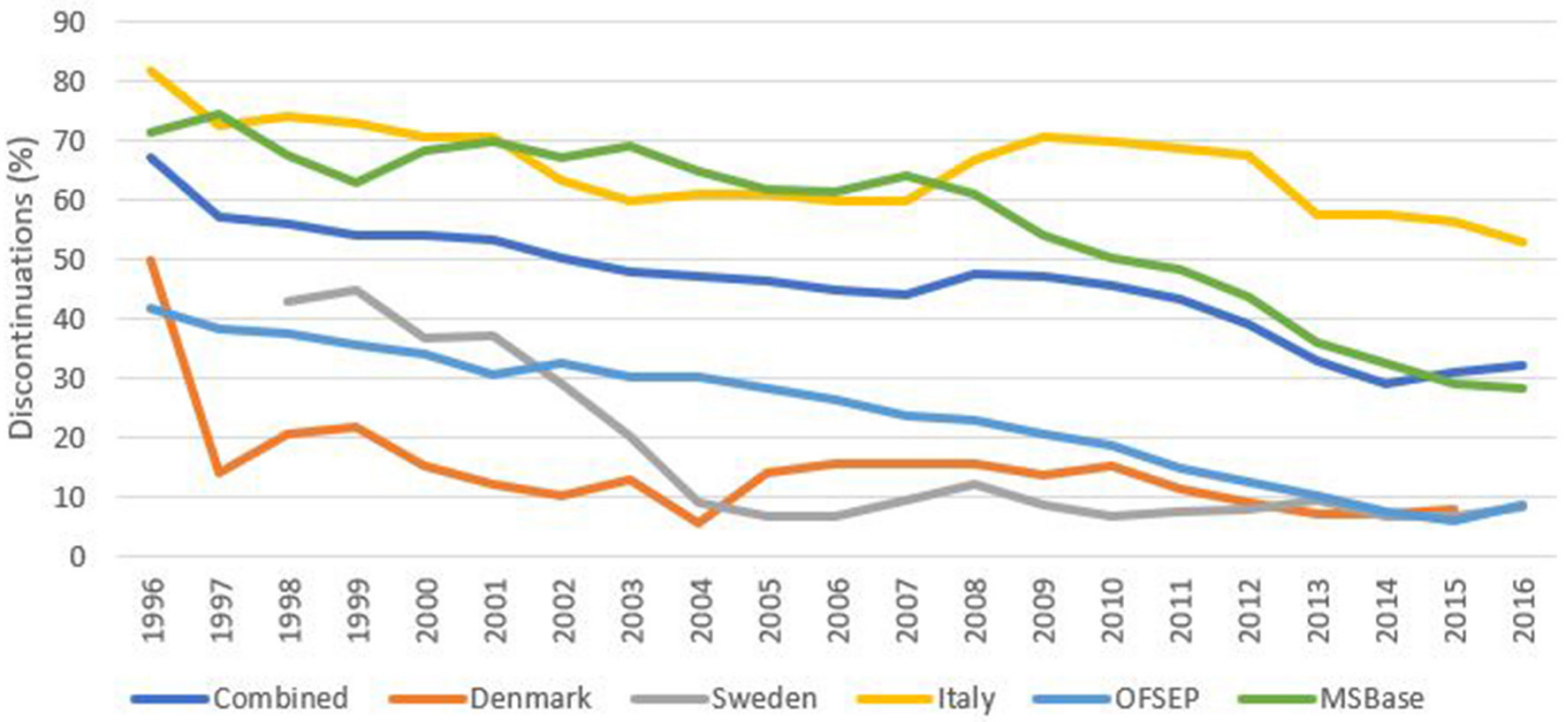
Percentage of discontinuations not reporting a discontinuation reason by registry.

**Figure 4:**
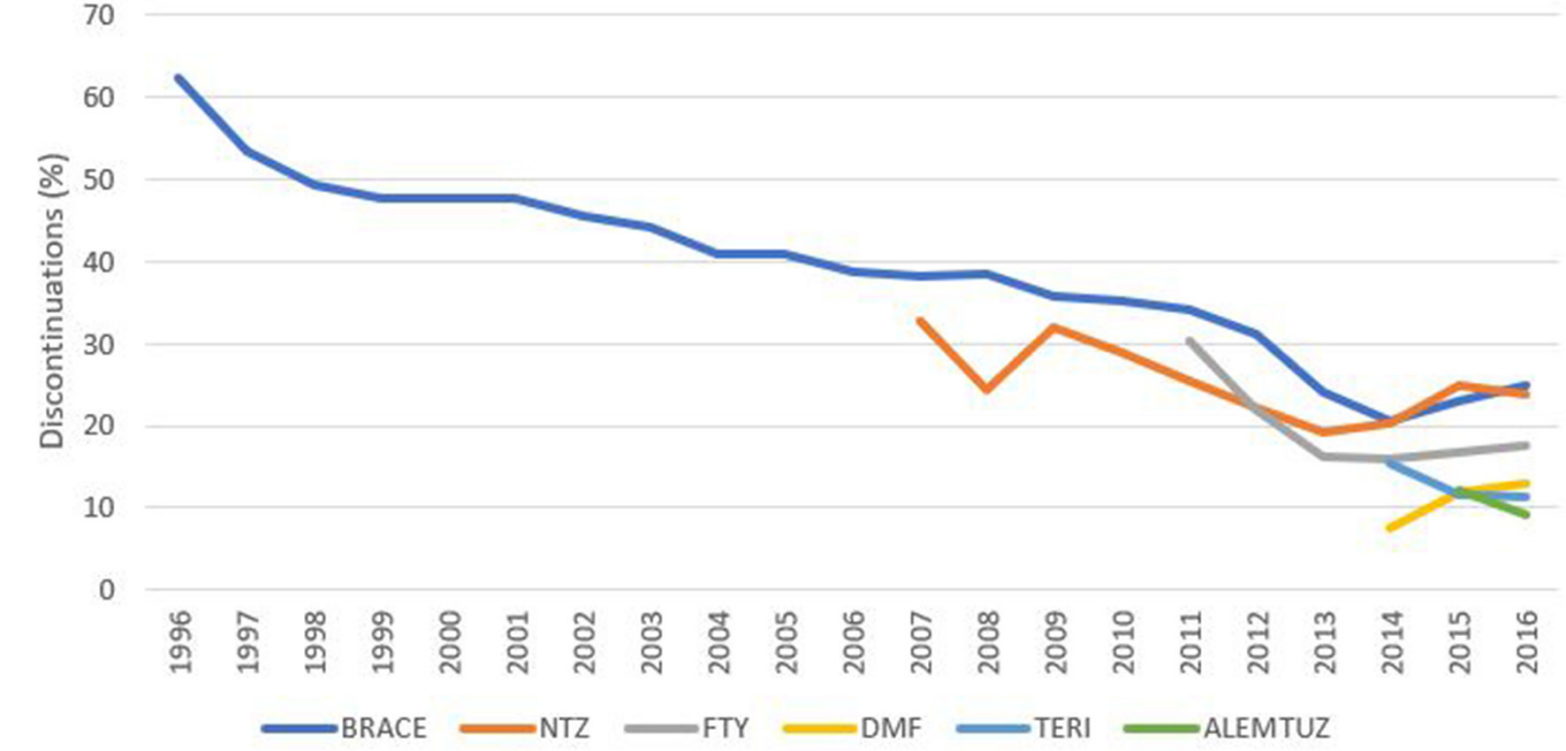
Percentage of discontinuations not reporting a discontinuation reason by DMT.

Where reason for treatment stopping was documented, lack of efficacy was the most frequently reported across the pooled data (23.2%), followed by adverse or side effects (16.1%) and intolerance (13.8%) across the full time period (Table 2). In terms of data quality, the proportion of treatment stops not reporting a reason for discontinuation had steadily declined across all registries from an average of 68% in 1996 down to just 32% in 2016 (Figure 3). There was however, some variability in this reduction by DMT/drug class with, for example, alemtuzumab associated with just 9% of discontinuations not reporting a reason in 2016, compared with 26% of the platform DMTs (Figure 4).

**Table 2:** Reason for discontinuation.

	**Category**	**Total**
Discontinuations		*n* = 184,013
Reason for discontinuation—*n* (%)	Inadequate efficacy	26,034 (14.1)
	Disease progression/EDSS progression/EDSS 7+	4,218 (2.3)
	Tolerability	15,491 (8.4)
	Adverse event/side effects/safety	18,145 (9.9)
	Allergic reaction	3,537 (1.9)
	Convenience	8,428 (4.6)
	Pregnancy (planned or confirmed), contraception cessation	6,733 (3.7)
	Scheduled stop	12,147 (6.6)
	Non-adherence/non-compliance/no motivation	4,555 (2.5)
	Development of neutralizing antibodies	1,273 (0.7)
	Deceased	266 (0.1)
	Secondary progressive MS	1,109 (0.6)
	Other	10,506 (5.7)
	Not reported/unknown	76,887 (41.8)

*EDSS, Expanded disability status scale; MS, Multiple sclerosis*.

## Discussion

Stopping disease-modifying treatment in MS is a common event, both across products and treatment epochs. Frequent treatment interruption, switching and discontinuation remains a major challenge for replicating the treatment efficacy observed in pivotal clinical trials in real-world clinical practice. In this descriptive analysis, we have compiled the largest ever dataset of MS-specific DMT treatment episodes to describe, quantify and illustrate patterns of discontinuation in an increasingly complex treatment environment. Thanks to standardization of variable definitions, and similarities in data structure between the leading MS registries contributing treatment data to the analysis, we were able to merge data files on almost 270,000 treatment episodes in just over 110,000 patients from five major MS registries. In this analysis, we focused the evolution of stopping, switching and discontinuation patterns over 20 years, from 1997 to 2016.

Perhaps the most striking feature of this descriptive analysis is large number of complex switching patterns and treatment pathways observed, with many patients moving from one DMT to another. The range and diversity of treatment pathways have multiplied over time as new treatments have entered the market. Disaggregating the analysis by the three most common treatment groups (IFN-β, glatiramer acetate and natalizumab), we observed greater complexity and diversity in treatment trajectories in patients initiating either first-line interferon-beta or glatiramer, relative to natalizumab. This is consistent with the longer time on the market of the two platform treatment groups. The early domination of the market by Interferon-β ensures these products dominate the discontinuation data over the observation period (15). However, the expansion of approved products for MS has led to a much more diverse discontinuation picture in the more recent years (Figure 1).

Regarding reasons for discontinuation, the pattern has been remarkably stable over the study period with lack of efficacy being the most common reason followed by lack of tolerance, side effects, convenience and disease progression in that order (Table 2 and Figure 2). However, these are likely to become more complex and more heterogenous as novel treatments and agents are added to the battery of available DMTs for MS (16, 17). Likewise, the proportion of patients continuing on a drug they are already prescribed has over the years been stable up until 2014 when a clear change took place which continued the following year to bring down overall continuation chance from 83 to 75%. This change may have been prompted by the introduction on the markets of novel per-oral drugs such as dimethyl fumarate and teriflunomide. Natalizumab, on the other hand, has shown a gradually decreased continuation rate each year after its introduction.

In spite of its size, this descriptive study has several limitations. Reason for discontinuation data was not reported for 41.8% of DMT stops. The amount of missing discontinuation reason data varied by registry, from a low of 10.5% of discontinuations in the Danish MS register, up to 63.5% in the Italian registry. Whilst this left a total of 107,126 DMT discontinuations with reasons reported to analyze, it remains a major limitation of this study, particularly as were limited to analyzing treatment stop reason to exactly how they were reported in each registry. However, discontinuation reason reporting greatly improved over the observation period and we would anticipate this trend would continue as the registries evolve further. Whilst overall there was good consistency in the type of discontinuation reason categories collected by each registry, there were some notable exceptions. Convenience was not available as a separate option in the Swedish registry and thus the convenience data is likely to represent an under-estimate of the reality. It also may explain why the “other reason” category was the most frequently reported discontinuation reason category in the Swedish data, being likely to include treatment switches made on the grounds of convenience. Similarly, ceasing treatment secondary to neutralizing antibodies was only present in the Danish and Swedish registries. There is also likely to be some overlap between related categories such as “lack of efficacy” and “disease progression,” or “adverse events,” “side effects,” and “lack of tolerance.” Furthermore, JCV status, an important determinate of pre-mature natalizumab discontinuation, was not sufficiently available to permit analysis or disaggregation. Finally, comparisons of proportions and percentages are strictly descriptive, with no statistical adjustment made for clinical or practice-based factors that may also influence persistence or discontinuation (8, 18, 19). Formal statistical comparisons of clinical outcomes by drug class or treatment pattern and sequence form, adjusted for patient and disease factors, the basis of a parallel study currently being undertaken by the study group. Future directions also included the formal analysis of drivers of, and outcomes from, different treatment switch patterns.

While our results show some changes in DMT stopping over time, likely reflecting the availability of alternative treatments, typically increasing over time, changes are mainly modest and gradual, suggesting that the main driver of change of treatment is likely to be properties of the various drugs, their formulations and routes of administration.

## Data Availability Statement

The availability of the datasets presented in this article are limited by the governing rules of the contributing registries that prohibit the sharing of both patient level data and aggregate data that may identify individual patients. Selected data and analyses may be accessed via direct request to the individual registries and subject to satisfying the data sharing permission rules of each registry.

## Ethics Statement

The studies involving human participants were reviewed and approved by each contributing registry to the Big MS Data Network according to their own ethics, operating and inclusion rules. Each registry is required to obtain their own approval prior to provision of the data and pooling.

## Author's Note

All authors/investigators from the Italian MS Registry, OFSEP and the MSBase Study Group are listed in the Supplementary Material.

## Author Contributions

TS was involved in the study design, data analysis, interpretation, manuscript preparation, and review. MM, PS, HB, AV, SV, MT, PI, FP, RH, LS, AM, and JH was involved in the study design, interpretation, and manuscript review. All authors contributed to the article and approved the submitted version.

## Conflict of Interest

TS received compensation for serving on scientific advisory boards, honoraria for consultancy and funding for travel from Biogen; speaker honoraria from Novartis. MM has served on scientific advisory board for Biogen Idec and Teva and has received honoraria for lecturing from Biogen Idec, Merck Serono, Sanofi-Aventis and Teva. MM has received support for congress participation from Biogen Idec, Merck Serono, Novartis and Genzyme. PS has served on scientific advisory boards for Merck Serono, Teva, Novartis, Sanofi-Aventis and Biogen Idec and has received research support from Biogen Idec, Novartis and Sanofi-Aventis and received speaker honoraria from Merck Serono, Novartis, Teva, Sanofi-Aventis, Biogen Idec and Genzyme. HB received compensation for serving on scientific advisory boards and as a consultant for Biogen, Novartis; speaker honoraria from Biogen Australia, Merck Serono Australia, Novartis Australia; travel support from Biogen Australia, Merck Serono Australia; research support from the CASS Foundation (Australia), Merck Serono Australia, the Royal Melbourne Hospital. SV received consulting and lecturing fees, travel grants and research support from Biogen, Celgene, Genentech, Genzyme, Medday pharmaceuticals, Merck Serono, Novartis, Roche, Sanofi Aventis, and Teva Pharma. MT has served on scientific Advisory Boards for Biogen, Novartis, Roche, and Genzyme; has received speaker honoraria and travel support from Biogen Idec, Sanofi-Aventis, Merck Serono, Teva, Genzyme and Novartis; and has received research grants for her Institution from Biogen Idec, Merck Serono, and Novartis. PI has served on scientific advisory boards for Biogen Idec, Bayer, Teva, Roche, Merck Serono, Novartis, and Genzyme and has received funding for travel and/or Speaker honoraria from Sanofi Aventis, Genzyme, Biogen Idec, Teva, Merck Serono, and Novartis. FP is an employee of Biogen. RH is an employee of Biogen and holds stock. JH has received honoraria for serving on advisory boards for Biogen, Sanofi-Genzyme, and Novartis and speaker's fees from Biogen, Novartis, Merck-Serono, Bayer-Schering, Teva, and Sanofi-Genzyme. JH has served as PI for projects, or received unrestricted research support from BiogenIdec, Merck-Serono, TEVA, Sanofi-Genzyme, and Bayer-Schering. JH MS research is funded by the Swedish Research Council and the Swedish Brain Foundation. The remaining authors declare that the research was conducted in the absence of any commercial or financial relationships that could be construed as a potential conflict of interest.
