# Supplementary material for: Treatment Switching and Discontinuation Over 20 Years in the Big Multiple Sclerosis Data Network
Source: Front Neurol. 2021 Mar 17;12:647811. doi: 10.3389/fneur.2021.647811 (PMC8010264; doi:10.3389/fneur.2021.647811)
Supplement: Supplementary file 1 [file Data_Sheet_1.pdf]

Observatoire Français de la Sclérose en Plaques (OFSEP) Co-investigators

Surname	First Name	Qualification	Affiliation
**Steering Committee**			
Casey	Romain	PhD	Observatoire français de la sclérose en plaques (OFSEP), Centre de coordination national, Lyon/Bron, France;
Cotton	François	MD	Hospices civils de Lyon, Hôpital Lyon sud, Service d'imagerie médicale et interventionnelle, Lyon/Pierre-Bénite, France;
De Sèze	Jérôme	MD	Hôpitaux universitaire de Strasbourg, Hôpital de Hautepierre, Service des maladies inflammatoires du système nerveux – neurologie, Strasbourg, France;
Douek	Pascal	MD	Union pour la lutte contre la sclérose en plaques (UNISEP), Ivry-sur-Seine, *France;*
Guillemin	Francis	MD	CIC 1433 Epidémiologie Clinique, Centre hospitalier régional universitaire de Nancy, Inserm et Université de Lorraine, Nancy, France;
Laplaud	David	MD	Centre hospitalier universitaire de Nantes, Hôpital nord Laennec, Service de neurologie, Nantes/Saint-Herblain, France;
Lebrun-Frenay	Christine	MD	Centre hospitalier universitaire de Nice, Université Nice Côte d'Azur, Hôpital Pasteur, Service de neurologie, Nice, France;
Dion	Armelle		Hospices civils de Lyon, Département de la recherche clinique et de l'innovation, Lyon, France;
Moreau	Thibault	MD	Centre hospitalier universitaire Dijon Bourgogne, Hôpital François Mitterrand, Service de neurologie, maladies inflammatoires du système nerveux et neurologie générale, Dijon, France;
Olaiz	Javier	PhD	Université Claude Bernard Lyon 1, Lyon ingéniérie projets, Lyon, France;
Pelletier	Jean	MD	Assistance publique des hôpitaux de Marseille, Centre hospitalier de la Timone, Service de neurologie et unité neuro-vasculaire, Marseille, France;
Rigaud-Bully	Claire		Fondation Eugène Devic EDMUS contre la sclérose en plaques, Lyon, France;
Stankoff	Bruno	MD	Assistance publique des hôpitaux de Paris, Hôpital Saint-Antoine, Service de neurologie, Paris, France
Vukusic	Sandra	MD	Hospices civils de Lyon, Hôpital Pierre Wertheimer, Service de neurologie A, Lyon/Bron, France
Zephir	Hélène	MD	Centre hospitalier universitaire de Lille, Hôpital Salengro, Service de neurologie, Lille, France
**Investigators**			
Marignier	Romain	MD	Hospices civils de Lyon, Hôpital Pierre Wertheimer, Service de neurologie A, Lyon/Bron, France;
Debouverie	Marc	MD	Centre hospitalier régional universitaire de Nancy, Hôpital central, Service de neurologie, Nancy, France;
Edan	Gilles	MD	Centre hospitalier universitaire de Rennes, Hôpital Pontchaillou, Service de neurologie, Rennes, France;
Ciron	Jonathan	MD	Centre hospitalier universitaire de Toulouse, Hôpital Purpan, Service de neurologie inflammatoire et neuro-oncologie, Toulouse, France;

Ruet	Aurélie	MD	Centre hospitalier universitaire de Bordeaux, Hôpital Pellegrin, Service de neurologie, Bordeaux, France;
Collongues	Nicolas	MD	Hôpitaux universitaire de Strasbourg, Hôpital de Hautepierre, Service des maladies inflammatoires du système nerveux – neurologie, Strasbourg, France;
Lubetzki	Catherine	MD	Assistance publique des hôpitaux de Paris, Hôpital de la Pitié-Salpêtrière, Service de neurologie, Paris, France;
Zephir	Hélène	MD	Centre hospitalier universitaire de Lille, Hôpital Salengro, Service de neurologie, Lille, France;
Labauge	Pierre	MD	Centre hospitalier universitaire de Montpellier, Hôpital Gui de Chauliac, Service de neurologie, Montpellier, France;
Defer	Gilles	MD	Centre hospitalier universitaire de Caen Normandie, Service de neurologie, Hôpital Côte de Nacre, Caen, France;
Cohen	Mikaël	MD	Centre hospitalier universitaire de Nice, Université Nice Côte d'Azur, Hôpital Pasteur, Service de neurologie, Nice, France;
Fromont	Agnès	MD	Centre hospitalier universitaire Dijon Bourgogne, Hôpital François Mitterrand, Service de neurologie, maladies inflammatoires du système nerveux et neurologie générale, Dijon, France;
Wiertlewsky	Sandrine	MD	Centre hospitalier universitaire de Nantes, Hôpital nord Laennec, Service de neurologie, Nantes/Saint-Herblain, France;
Berger	Eric	MD	Centre hospitalier régional universitaire de Besançon, Hôpital Jean Minjoz, Service de neurologie, Besançon, France;
Clavelou	Pierre	MD	Centre hospitalier universitaire de Clermont-Ferrand, Hôpital Gabriel-Montpied, Service de neurologie, Clermont-Ferrand, France;
Audoin	Bertrand	MD	Assistance publique des hôpitaux de Marseille, Centre hospitalier de la Timone, Service de neurologie et unité neuro-vasculaire, Marseille, France;
Giannesini	Claire	MD	Assistance publique des hôpitaux de Paris, Hôpital Saint-Antoine, Service de neurologie, Paris, France;
Gout	Olivier	MD	Fondation Adolphe de Rothschild de l'œil et du cerveau, Service de neurologie, Paris, France;
Thouvenot	Eric	MD	Centre hospitalier universitaire de Nîmes, Hôpital Carémeau, Service de neurologie, Nîmes, France;
Heinzlef	Olivier	MD	Centre hospitalier intercommunal de Poissy Saint-Germain-en-Laye, Service de neurologie, Poissy, France;
Al-Khedr	Abdullatif	MD	Centre hospitalier universitaire d'Amiens Picardie, Site sud, Service de neurologie, Amiens, France;
Bourre	Bertrand	MD	Centre hospitalier universitaire Rouen Normandie, Hôpital Charles-Nicolle, Service de neurologie, Rouen, France;
Casez	Olivier	MD	Centre hospitalier universitaire Grenoble-Alpes, Site nord, Service de neurologie, Grenoble/La Tronche, France;
Cabre	Philippe	MD	Centre hospitalier universitaire de Martinique, Hôpital Pierre Zobda-Quitman, Service de Neurologie, Fort-de-France, France;
Montcuquet	Alexis	MD	Centre hospitalier universitaire Limoges, Hôpital Dupuytren, Service de neurologie, Limoges, France;

Wahab	Abir	MD	Assistance publique des hôpitaux de Paris, Hôpital Henri Mondor, Service de neurologie, Créteil, France;
Camdessanché	Jean-Philippe	MD	Centre hospitalier universitaire de Saint-Étienne, Hôpital Nord, Service de neurologie, Saint-Étienne, France;
Bakchine	Serge	MD	Centre hospitalier universitaire de Reims, Hôpital Maison-Blanche, Service de neurologie, Reims, France;
Maurousset	Aude	MD	Centre hospitalier régional universitaire de Tours, Hôpital Bretonneau, Service de neurologie, Tours, France;
Ben Nasr	Haifa	MD	Centre hospitalier sud francilien, Service de neurologie, Corbeil-Essonnes, France;
Hankiewicz	Karolina	MD	Centre hospitalier de Saint-Denis, Hôpital Casanova, Service de neurologie, Saint-Denis, France;
Pottier	Corinne	MD	Centre hospitalier de Pontoise, Service de neurologie, Pontoise, France;
Maubeuge	Nicolas	MD	Centre hospitalier universitaire de Poitiers, Site de la Milétrie, Service de neurologie, Poitiers, France;
Labeyrie	Céline	MD	Assistance publique des hôpitaux de Paris, Hôpital Bicêtre, Service de neurologie, Le Kremlin-Bicêtre, France;
Nifle	Chantal	MD	Centre hospitalier de Versailles, Hôpital André-Mignot, Service de neurologie, Le Chesnay, France;
