# Supplementary material for: Treatment Switching and Discontinuation Over 20 Years in the Big Multiple Sclerosis Data Network
Source: Front Neurol. 2021 Mar 17;12:647811. doi: 10.3389/fneur.2021.647811 (PMC8010264; doi:10.3389/fneur.2021.647811)
Supplement: Supplementary file 2 [file Data_Sheet_2.pdf]

MSBase Study Group Investigators

Investigator	Affiliation
Eva Havrdova	General University Hospital
Serkan Ozakbas	Dokuz Eylul University
Vahid Shaygannejad	Al-Zahra Hospital, Isfahan University of Medical Sciences
Rana Karabudak	Hacettepe University
Raed Alroughani	Amiri Hospital
Rocio Lopez Ruiz, Sara Eichau	Hospital Universitario Virgen Macarena
Cavit Boz	KTU Medical Faculty Farabi Hospital
Murat Terzi	Mayis University, Medical Faculty
Pierre Duquette	CHUM - Hopital Notre Dame
Francois Grand Maison	Neuro Rive-Sud
Pierre Grammond	Centre de réadaptation déficience physique Chaudière-Appalache
Ayse Altintas	Koc University School of Medicine
Allan Kermode	Perron Institute for Neurological and Translational Science
Tomas Kalinck	The Royal Melbourne Hospital
Recai Turkoglu	Haydarpasa Numune Training and Research Hospital
Aysun Soysal	Bakirkoy Education and Research Hospital for Psychiatric and Neurological Diseases
Katherine Buzzard & Olga Skabina	Box Hill Hospital
Jeannette Lechner Scott	John Hunter Hospital
Seyed Aidin Sajedi	Golestan
Yolanda Blanco	Hospital Clinic de Barcelona
Oliver Gerlach	Maaslandziekenhuis
Vincent Van Pesch	Cliniques Universitaires Saint-Luc
Stella Hughes	Belfast HSC Trust
Anneke Van der Walt	Alfred Health
Celia Oreja-Guevara	Hospital Universitario La Paz
Christopher McGuigan	St Vincent's University Hospital
Michael Barnett	Brain and Mind Centre (BMRI)
Cristina Ramo	Hospital Germans Trias i Pujol
Jens Kuhle	Universitatsspital Basel
Riadh Gouider	Razi hospital
Bhim Singhal	Bombay Hospital Institute of Medical Sciences
Suzanne Hodgkinson	Liverpool Hospital
Edgardo Cristiano & Juan Ignacio Rojas	Hospital Italiano
Radek Ampapa	Nemocnice Jihlava
Ernest Butler	Monash Medical Centre
Mark Slee	Flinders Medical Centre
Julie Prevost	CSSS Saint-Jérôme
Bruce Taylor	Royal Hobart Hospital
Pamela McCombe	St Andrews Place
Thor Petersen	Kommunehospitalet
Koen de Gans	Groene Hart ziekenhuis
Erik Van Munster	Jeroen Bosch Ziekenhuis
Bart Van Wijmeersch	Rehabilitation and MS-Centre Overpelt

Guy Laureys	University Hospital Ghent
LGF Sinnige	Medical Center Leeuwarden
Richard Macdonell	Austin Health
Dheeraj Khurana	PGIMER
Ricardo Fernandes Bolanos	Hospital Universitario Virgen de Valme
Jose Luis Sanchez Menoyo	Hospital de Galdakao-Usansolo
Maria Edite Rio	Hospital São João
Steve Vucic	Westmead Hospital
Jihad Inshasi	RASHID HOSPITAL
Tamara Castillo Trivino	Hospital Donostia
Orla Gray	South East Trust
Jamie Campbell	Craigavon Area Hospital
Pamela McCombe	Royal Brisbane and Women's Hospital
Maria Laura Sladino	Ineba
Talal Al-Harbi	King Fahad Specialist Hospital-Dammam
Dieter Poehlau	Multiple Sclerosis Centre Kamillus-Klinik
Ilya Kister	New York University School of Medicine
Leontien Den Braber-moerland	Francicus Ziekenhuis
Yara Fragoso	Universidade Metropolitana de Santos
Cameron Shaw	Geelong Hospital
Norma Deri	Hospital Fernandez
Patrice Lalive	Geneva University Hospital
Niel Shuey	St Vincents Hospital, Fitzroy
Norbert Vella	Mater Dei Hospital
Walter Oleschko Arruda	Hospital Ecoville
Angel Perez Sempere	Hospital General Universitario de Alicante
Tunde Csepany	University of Debrecen
Shlomo Fletcher	Assaf Harofeh Medical Center
Fraser Moore	Jewish General Hospital
John Parratt	Royal North Shore Hospital
Danny Decoo	AZ Alma
Todd Hardy	Concord Repatriation General Hospital
Madgolna Simo	Semmelweis University Budapest
Gabor Lovas	Jahn Ferenc Teaching Hospital
Tatjana Petkovska-Boskova	Clinic of Neurology Clinical Center
Eli Skromne	Hospital Angeles de las Lomas. Instituto Mexicano de Neurociencias.
Donald McCarren	Thomas Jefferson University
Jabir Alkhaboori	Royal Hospital
Jose Andres Dominiuez	HOSPITAL UNIVERSITARIO DE LA RIBERA
Karim Kotkata	Alexandria University Student Hospital
Joyce Pauline Joseph	HOSPITAL KUALA LUMPUR
Elizabeth Alejandra Bacile Bacile	Instituto de Neurociencias Cordoba
Vetere Santiago	HIGA Gral. San Martin LaPlata
Carlos Vrech	Sanatorio Allende
Kristina Kovacs	Péterfy Sandor Hospital
Mike Boggild	The Townsville Hospital
Ik Lin Tan	Macquarie University Hospital

Alfredo Firstenfeld	Clinica Modelo de Lanus
Piroska Imre	Veszprém Megyei Csolnoky Ferenc Kórház zrt.
Jyh Yung	Penang General Hospital
Stephane Charest	CHRTR
Alejandro Jose Diaz Jimenez	Instituto de Seguridad Social de Guatemala
Lucienne Costa-Frossard Franca	RAMON Y CAJAL UNIVERSITY HOSPITAL
Carolyn Young	The Walton Centre for Neurology and Neurosurgery
Tunde Erdelyi	Josa András Hospital
Attila Sas	BAZ County Hospital
Eniko Dobos	Szent Imre Hospital
Vladimir Bojkovski	Clinical Centar-Neurology
Shereen Fathi	Maadi MS Center
Cecilia Rajda	University of Szeged
Alejandro Caride	HOSPITAL DE CLINICAS
Magda Tsolaki	G. Papanicolaou Hospital
Gabor Rum	Petz A. County Hospital
Anita Trauninger	University of Pécs, Medical School
Karyn Boundy	Western Neurology
Istvan Deme	Kaposi Mór Teaching Hospital
Carmen-Adella Sirbu	Central Clinical Emergency Military Hosp.
Benjamin Greenberg	UT Southwestern
Marcos Burgos	HOSPITAL SAN BERNARDO
Ulku Turk Boru	Dr Lutfi Kirdar Kartal Education and Research Hosp.
Kai Chen Wang	Cheng Hsin General Hospital, Taipei,Taiwan
Pei Chiek The	Hospital Tuanku Ja'afar Seremban
