# Supplementary material for: Treatment Switching and Discontinuation Over 20 Years in the Big Multiple Sclerosis Data Network
Source: Front Neurol. 2021 Mar 17;12:647811. doi: 10.3389/fneur.2021.647811 (PMC8010264; doi:10.3389/fneur.2021.647811)
Supplement: Supplementary file 3 [file Data_Sheet_3.pdf]

Italian MS Registry Co-investigators

Name	Affiliation
**Francesco Passantino**, ***MD***	Azienda Ospedaliera SS. Antonio e Biagio e Cesare Arrigo, Divisione Di Neurologia, Alessandria, Italia
**Maura Chiara Danni,** ***MD***	Ospedali Riuniti di Ancona, Centro SM - Clinica Neurologica, Ancona, Italia
**Susanna Cordera,** ***MD***	Ospedale Regionale della Valle D'Aosta, Struttura Complessa di Neurologia, Italia
**Gabriella Cacchio',** ***MD***	Ospedale C. e G. Mazzoni, Ambulatorio dedicato Sclerosi Multipla, UO Neurologia AV5, Ascoli Piceno, Italia
**Michele Ragno,** ***MD***	Ospedale Civile Madonna del Soccorso, Ambulatorio Sclerosi Multipla, UO di Neurologia AV5, San Benedetto del Tronto, Italia
**Rocco Totaro,** ***MD***	Ospedale San Salvatore, Centro Malattie Demielinizzanti, Clinica Neurologica, L'Aquila, Italia
**Sauro Severi,** ***MD***	Ambulatorio SM - Sezione Neurologia, Ospedale Valdarno, Montevarchi (AR), Italia
**Daniele Spitaleri,** ***MD***	AORN San G. Moscati Centro Sclerosi Multipla, U.O.C. Neurologia, Avellino, Italia
**Maurizia Gatto,** ***MD***	Ospedale Generale Regionale F. Miulli Centro Malattie Demielinizzanti, Bari, Italia
**Damiano Paolicelli,** ***MD***	Centro SM, Dipartimento di Scienze Mediche di Base, Neuroscienze ed Organi di Senso Università di Bari, Italia
**Bonaventura Ardito,** ***MD***	Ospedale Della Murgia Fabio Perinei, Centro Sclerosi Multipla, Altamura, Italia
**Valeria Barcella,** ***MD***	Ospedale Papa Giovanni XXIII, Centro Provinciale Sclerosi Multipla, USS Malattie Autoimmuni, Bergamo, Italia
**Lorenzo Capone,** ***MD***	MD, Ospedale degli Infermi, Centro Clinico delle Malattie Demielinizzanti dell'ASL di Biella, Biella, Italia
**Piero Nicolao,** ***MD***	Ospedale Di Feltre, Reparto Di Neurologia, ULSS 1 Dolomiti, Feltre, Italia
**Alessandra Lugaresi,** ***MD***	UOSI Riabilitazione Sclerosi Multipla IRCCS – ISNB, Bologna, Italia
**Augusto Rini,** ***MD***	Ospedale A. Perrino, Centro Sclerosi Multipla, Brindisi, Italia
**Vincenzo Sidoti, MD**	Azienda Socio Sanitaria Territoriale (A.S.S.T.) della Franciacorta, Italia
**Marta Bianchi,** ***MD***	Ospedale Di Esine, Reparto di Neurologia, Esine, Italia
**Imma Plasmati,** ***MD***	P.O. Dimiccoli, Centro SM c/o U.O. di Neurologia, Barletta, Italia
**Eleonora Cocco,** ***MD***	P.O. Binaghi, Centro Regionale Per La Diagnosi E La Cura Della Sclerosi Multipla, Cagliari, Italia
**Onofrj Marco,** ***MD***	MD, Università G. D'Annunzio, Policlinico SS Annunziata Clinica Neurologica, Chieti, Italia
**Fiorella Mondino,** ***MD***	ASO S.Croce e Carle, Centro Sclerosi Multipla, SC Neurologia, Cuneo, Italia
**Alessia Di Sapio,** ***MD***	Ospedale Regina Montis Regalis, Centro SM, Mondovì, Italia
**Raffaella Clerici,** ***MD***	Ospedale Generale di zona Valduce, Centro ad Alta Specializzazione per la diagnosi e la cura della sclerosi multipla, Como, Italia
**Nerina Mascoli,** ***MD***	ASST Lariana Ospedale S. Anna, Centro SM UO Neurologia, Como, Italia
**Maria Teresa Ferrò,** ***MD***	Ospedale Maggiore di Crema, Neuroimmunologia - Centro Provinciale per la diagnosi e terapia della Sclerosi Multipla, Crema, Italia
**Roberto Bruno Bossio,** ***MD***	ASP di Cosenza, U.O. di Neurologia, Centro SM, Cosenza, Italia
**Francesco Patti,** ***MD***	AOL Policlinico Vittorio Emanuele - Università di Catania, Centro Sclerosi Multipla, Catania, Italia
**Davide Maimone,** ***MD***	Ospedale Garibaldi, Centro Sclerosi Multipla, Nesima, Italia
**Paola Valentino,** ***MD***	Policlinico Universitario- Campus Germaneto, Centro Sclerosi Multipla, Catanzaro, Italia
**Silvia Strumia,** ***MD***	Ambulatorio Sclerosi Multipla della U.O. di Neurologia, AUSL della Romagna, Forlì, Italia
**Maura Pugliatti,** ***MD***	Centro Di Servizio E Ricerca Sulla Sclerosi Multipla AOU Di Ferrara, Italia
**Luisa Maria Caniatti,** ***MD***	Azienda ospedale università S. Anna di Ferrara - Cona Ferrara, U.O.di Neurologia, Ferrara, Italia
**Carlo Avolio,** ***MD***	Centro Interdipartimentale Malattie Demielinizzanti - AOU Ospedali Riuniti Di Foggia, Italia
**Paola Crociani,** ***MD***	IRCCS Casa Sollievo Della Sofferenza, Centro SM, SC Neurologia, S. Giovanni Rotondo, Italia
**Maria Pia Amato, MD**	Dipartimento NEUROFARBA Sezione Neuroscienze, Università Degli Studi Di Firenze, Centro SM Neurologia 1, AOU Careggi, Firenze, Italia
**Luca Massacesi,** ***MD***	Centro di riferimento regionale per il trattamento della sclerosi multipla, SOD Neurologia II, AOU Careggi, Dipartimento Neuroscienze - Università di Firenze, Italia
**Susanna Malagù,** ***MD***	Ospedale Bufalini, Centro Sclerosi Multipla, U.O. di Neurologia, Cesena, Italia

**Giuseppe Ribizzi,** ***MD***	MD, Ospedale Policlinico San Martino, U.O. Neurologia, Dipartimento Di Neuroscienze E Organi Di Senso, Genova, Italia
**Matilde Inglese,** ***MD***	Ospedale Policlinico San Martino (DiNOGMI), Centro Per Lo Studio E La Cura Della Sclerosi Multipla E Malattie Demielinizzanti - Dipartimento Di Neuroscienze, Riabilitazione, Oftalmologia, Genetica E Scienze MaternoInfantili, Clinica Neurologica, Genova, Italia
**Simonetta Venturi,** ***MD***	E.O. Ospedale Galliera, Ambulatorio Sclerosi Multipla, Genova, Italia
**Paola Gazzola,** ***MD***	SC Neurologia, Ospedale P. Antero Micone, Ambulatorio Sclerosi Multipla - Neurologia ASL 4 Chiavarese, Genova, Italia
**Giampaolo Brichetto,** ***MD***	Servizio di Riabilitazione AISM Liguria, Italia
**Roberto Marconi,** ***MD***	Ospedale Misericordia, U.O. Neurologia, Grosseto, Italia
**Paolo Bellantonio,** ***MD***	Centro Sclerosi Multipla - IRCCS Neuromed, Pozzilli, Italia
**Roberto Balgera,** ***MD***	MD, Azienda Ospedaliera A. Manzoni, Centro Sclerosi Multipla, Divisione di Neurologia, Lecco, Italia
**Francesca De Robertis,** ***MD***	Ospedale Vito Fazzi, Divisione di Neurologia, Lecce, Italia
**Silvia Fermi,** ***MD***	Ospedale Maggiore di Lodi, Div. Neurologia, Lodi, Italia
**Franco Fausto,** ***MD***	Ospedale della Versilia, Ambulatorio Sclerosi Multipla, Italia
**Monica Mazzoni,** ***MD***	Ospedale San Luca, Centro Malattie Disimmuni Del SNC E SNP, Lucca, Italia
**Giuseppe Meucci,** ***MD***	Spedali Riuniti di Livorno, Ambulatorio Sclerosi Multipla, Unità Operativa di Neurologia e Neurofisiopatologia, Livorno, Italia
**Maria Gabriella Coniglio,** ***MD***	P.O. Madonna delle Grazie, Centro Sclerosi Multipla, Matera, Italia
**Guido Cavaletti,** ***MD***	Centro Di Neuroimmunologia, Ospedale S. Gerardo, Monza, Italia
**Elisabetta Cartechini,** ***MD***	Ospedale di Macerata, Centro Sclerosi Multipla - c/o UOC Neurologia, Macerata, Italia
**Maria Buccafusca,** ***MD***	A.O.U. Policlinico Martino, Centro Sclerosi Multipla, Messina, Italia
**Placido Bramanti,** ***MD***	IRCCS Centro Neurolesi Bonino Pulejo, Messina, Italia
**Massimo Filippi,** ***MD***	Ospedale San Raffaele, Centro Sclerosi Multipla, Milano, Italia
**Marco Rovaris,** ***MD***	IRCCS Fondazione Don Carlo Gnocchi, Centro SM, Italia
**Marco Ronzoni,** ***MD***	ASST-Rhodense, Garbagnate Milanese, Centro SM, Italia
**Valentina Torri Clerici,** ***MD***	Fondazione IRCCS Istituto Neurologico Carlo Besta, Centro Sclerosi Multipla, Milano, Italia
**Luca Chiveri,** ***MD***	ASST Ovest Milanese, Centro SM, Dipartimento Di Neuroscienze, Legnano, Italia
**Pierluigi Bertora,** ***MD***	ASST FBF SACCO P.O. L. Sacco, Centro Sclerosi Multipla, UO Neurologia, Milano, Italia
**Simone Tonietti,** ***MD***	Ospedale S. Carlo Borromeo, Centro Sclerosi Multipla - UOC Neurologia, Milano, Italia
**Elio Scarpini,** ***MD***	Ospedale Policlinico, Università Di Milano, Centro SM, U.O. Mal. Neurodegenerative E Demielinizzanti, Milano, Italia
**Alessandra Protti,** ***MD***	ASST Grande Ospedale Metropolitano Niguarda, Milano, Italia
**Patrizia Sola,** ***MD***	Azienda Ospedaliero-Universitaria/OCSAE, UO Neurologia, Centro Malattie Demielinizzanti, Dipartimento Di Neuroscienze, Università di Modena E Reggio Emilia, Modena, Italia
**Diana Ferraro,** ***MD***	Azienda Ospedaliera Universitaria, Modena, Italy
**Mario Santangelo,** ***MD***	Ospedale Di Carpi, U.O. C. Di Neurologia, Modena, Italia
**Carlo Maremmani, MD**	Civico Ospedale di Carrara, Centro Sclerosi Multipla, Divisione Neurologica, Carrara, Italia
**Giacomo Lus,** ***MD***	Centro Clinico per la Sclerosi Multipla, II Clinica Neurologica, II Università di Napoli, Italia
**Antonio Gallo,** ***MD***	Università Degli Studi Della Campania 'Luigi Vanvitelli', I Clinica Neurologica, Napoli, Italia
**Giorgia Teresa Maniscalco,** ***MD***	Ospedale A. Cardarelli, Centro regionale SM, Napoli, Italia
**Vincenzo Brescia Morra,** ***MD***	AOU Policlinico Federico II, Centro Regionale Sclerosi Multipla, Unità Operativa Semplice Neurologia, Napoli, Italia
**Giuseppe Orefice,** ***MD***	AOU Policlinico Federico II, Centro Provinciale Sclerosi Multipla, Napoli, Italia
**Leonardo Sinisi,** ***MD***	Ospedale San Paolo, Centro SM, UOC Di Neurologia, Napoli, Italia
**Cristoforo Comi,** ***MD***	AOU Maggiore della Carità, Centro Sclerosi Multipla, Clinica Neurologica, Università Piemonte Orientale, Novara, Italia
**Maria Luisa Piras,** ***MD***	Ospedale S. Francesco - Centro diagnosi, cura e ricerca per Sclerosi Multipla, Nuoro, Italia
**Giuseppe Salemi,** ***MD***	Centro Per La Diagnosi E Cura Della SM E Delle Malattie Demielinizzanti – Dipartimento Emergenza, Urgenza E Neuroscienze, Università Di Palermo, Palermo, Italia
**Salvatore Cottone, MD**	Centro Di Riferimento Regionale Per La Malattie Neuroimmunologiche Dell'A.O.O.R. Villa Sofia-Cervello, Palermo, Italia

**Luigi M. E. Grimaldi, *MD***	Fondazione Istituto G. Giglio - Centro SM, Cefalù, Italia
**Giuseppe Santangelo, *MD***	Ospedale Pediatrico G. di Cristina, Centro regionale Sclerosi Multipla in età evolutiva, Palermo, Italia
**Paolo Gallo, *MD***	Azienda Ospedaliera-Università degli Studi Di Padova, Centro Specializzato Regionale per la Sclerosi Multipla (CeSMuV), Dipartimento Di Neuroscienze DNS, Padova, Italia
**Francesco D'Andrea, *MD***	Casa Di Cura Villa Serena, Centro SM, UO Neurologia, Pescara, Italia
**Francesco Corea, *MD***	Ospedale San Giovanni Battista, Ambulatorio malattie infiammatorie demielinizzanti, Foligno, Italia
**Paola Sarchielli, *MD***	Ospedale S. Maria della Misericordia, Centro Malattie Demielinizzanti, Perugia, Italia
**Maria Grazia Celani, *MD***	Servizio Per Le Malattie Demielinizzanti, SC Di Neurofisiopatologia-Azienda Ospedaliera Di Perugia, Italia
**Cristina Frittelli, *MD***	Ospedale Lotti, Ambulatorio Malattie Demielinizzanti, UOC Neurologia, Pontedera, Italia
**Livia Pasquali, *MD***	Centro Malattie Demielinizzanti UO Neurologia, Dipartimento Di Medicina Clinica E Sperimentale, Università Di Pisa, Italia
**Mario Falcini, *MD***	Ospedale di Prato, Centro per la Sclerosi Multipla, Unità Operativa di Neurologia, Prato, Italia
**Franco Granella, *MD***	Centro Sclerosi Multipla, Azienda Ospedaliero-Universitaria di Parma, Italia
**Ilaria Pesci, *MD***	Ospedale Di Vaio, Centro SM, UO Neurologia, Fidenza, Italia
**Roberto Bergamaschi, *MD***	S.S. Sclerosi Multipla dell'IRCCS Fondazione Istituto Neurologico Nazionale C. Mondino, Pavia, Italia
**Diomira Acquistapace, *MD***	Azienda Ospedaliera Regionale S. Carlo, Centro Sclerosi Multipla, Potenza, Italia
**Umberto Aguglia, *MD***	Grande Ospedale Metropolitano Bianchi Melacrino Morelli, Ambulatorio Sclerosi Multipla, Reggio Calabria, Italia
**Sara Montepietra, *MD***	U.O.C. Neurologia, Ospedaliera Santa Maria Nuova, Centro SM, Reggio Emilia, Italia
**Antonello Giordano, *MD***	P.O. R. Guzzardi, S.C. Provinciale di Neurologia, ASP Ragusa, Italia
**Mario Di Napoli, *MD***	Ospedale San Camillo De Lellis, Centro Sclerosi Multipla, U.O. di Neurologia, Rieti, Italia
**Marco Salvetti, *MD***	CENTERS Centro Neurologico Terapie Sperimentali, Sapienza Università Di Roma, Azienda Ospedaliera S. Andrea, Roma, Italia
**Carlo Pozzilli, *MD***	Policlinico S. Andrea, Centro SM, Università Sapienza Roma, Italia
**Massimiliano Mirabella, *MD***	Fondazione Policlinico Universitario A. Gemelli IRCCS, UO Sclerosi Multipla, Università Cattolica Del Sacro Cuore, Roma, Italia
**Antonella Conte, *MD***	Policlinico Umberto I, Università Sapienza Roma, Italia
**Claudio Gasperini, *MD***	Azienda Osp. S. Camillo Forlanini, Centro Sclerosi Multipla, Roma, Italia
**Marco Peresson, *MD***	Ospedale Fatebenefratelli San Pietro, Centro Clinico Sclerosi Multipla, Roma, Italia
**Maria Grazia Grasso, *MD***	Fondazione S. Lucia, Centro Sclerosi Multipla, Roma, Italia
**Elisabetta Ferraro, *MD***	PO San Filippo Neri - ASL Roma 1, Roma, Italia
**Vincenzo Di Lazzaro, *MD***	Policlinico Universitario Campus Bio-Medico, Centro SM, UOC Di Neurologia, Roma, Italia
**Girolama Marfia, *MD***	Policlinico Università di Roma Tor Vergata, UOSB Centro di Riferimento Regionale per la Sclerosi Multipla, Roma, Italia
**Daniela de Pascalis, *MD***	Ospedale S. Eugenio, Centro regionale per la diagnosi e cura della Sclerosi Multipla e malattie demielinizzanti, Roma, Italia
**Carlo Piantadosi, *MD***	Az. Ospedaliera S. Giovanni-Addolorata, Centro Sclerosi Multipla, Roma, Italia
**Massimiliano Valeriani, *MD***	Ospedale Bambino Gesù, Centro per la diagnosi e la cura delle malattie infiammatorie demielinizzanti in età pediatrica, Roma, Italia
**Vincenzo Busillo, *MD***	Ospedale Maria SS. Addolorata - Centro Diagnosi e Terapia Sclerosi Multipla, Salerno, Italia
**Paolo Barone, *MD***	Azienda Ospedaliera San Giovanni di Dio e Ruggi d'Aragona, Divisione Neurologica, Salerno, Italia
**Monica Ulivelli, *MD***	UOS Neuroimmunologia clinica, Ambulatorio Sclerosi multipla AOUS, Siena, Italia
**Nicola De Stefano, *MD***	Azienda Ospedaliera Universitaria Senese, Ambulatorio Sclerosi multipla - U.O.S.A. Malattie Neurodegenerative e Demielinizzanti, Siena, Italia
**Giuseppe Santuccio, *MD***	Azienza Socio Sanitaria Territoriale (ASST) della Valtellina e Alto Lario, reparto di Neurologia, Sedi di Sondrio e Sondalo, Italia
**Sergio Parodi, *MD***	Ospedale Civile S. Andrea, Centro Sclerosi Multipla e Malattie Demielinizzanti, La Spezia, Italia
**Antonio Cappellani, *MD***	Ambulatorio di Neurologia, Centro Sclerosi Multipla, Ex Ospedale Neuropsichiatrico, Siracusa, Italia

**Sebastiano Traccis, *MD***	Presidio Ospedaliero di Ozieri, Centro Prescrittore Sclerosi Multipla, U.O. di Neurologia, Sassari, Italia
**Roberto Zarbo, *MD***	Azienda Ospedaliero-Universitaria Di Sassari, Centro Per La Diagnosi E Cura Della SM, Sassari, Italia
**Tiziana Tassinari, *MD***	Ospedale Santa Corona, Centro SM, S.C. Neurologia, Pietra Ligure, Italia
**Fabio Bandini, *MD***	Ospedale S. Paolo, Divisione di Neurologia, Savona, Italia
**Annamaria Marson, *MD***	Ospedale Civile, Neurologia BC Chivasso-Ivrea, Italia
**Paola Cavalla, *MD***	Città Della Salute E Della Scienza Di Torino, Centro SM, Neurologia 1 D.U., Torino, Italia
**Marinella Clerico, *MD***	Azienda Ospedaliero Universitaria San Luigi Gonzaga, S.C.D.U. di Neurologia 1, Orbassano, Italia
**Giulia De Rosa, *MD***	Ospedale Civile, Centro Sclerosi Multipla, Divisione Di Neurologia, Ivrea, Italia
**Antonio Bertolotto, *MD***	AOU San Luigi, Centro Di Riferimento Regionale Per La SM (CRESM), SCDO Neurologia, Orbassano, Italia
**Daniele Imperiale, *MD***	Ospedale Maria Vittoria, Centro Sclerosi Multipla, Divisione di Neurologia, Torino, Italia
**Marika Vianello, *MD***	Ospedale Regionale Cà Fancello, Centro Sclerosi Multipla UO, UO Neurologia, Treviso, Italia
**Bruno Marini, *MD***	Ospedale S. Giacomo Apostolo, U.O.C. Neurologia, ULSS2- Marca Trevigiana-Regione Veneto, Castelfranco Veneto, Italia
**Marianna Fortunato, *MD***	Ospedale Civile di Conegliano Veneto, Ambulatorio Malattie Demielinizzanti, Unità Operativa Complessa di Neurologia, Conegliano Veneto, Italia
**Daniela Cargnelutti, MD**	ASUIUD P.O. S. Maria Della Misericordia, SOC Neurologia - Day Hospital, Udine, Italia
**Mauro Zaffaroni, *MD***	Ospedale Di Gallarate, Centro Sclerosi Multipla, ASST Della Valle Olona, Gallarate, Italia
**Davide Nasuelli, *MD***	Presidio Ospedaliero Di Saronno ASST Della Valle Olona Ambulatorio Sclerosi Multipla, Saronno, Italia
**Paola Banfi, *MD***	Ospedale di Circolo e Fondazione Macchi, Centro Sclerosi Multipla, Ambulatorio Malattie Demielinizzanti, Varese, Italia
**Andrea Mauro Brioschi, *MD***	Istituto Auxologico Italiano IRCCS, Istituto Scientifico Ospedale S. Giuseppe e Ambulatorio, Milano, Italia
**Rocco Quatrale, *MD***	Ospedale dell'Angelo, Ambulatorio Sclerosi Multipla, Divisione di Neurologia, Venezia, Italia
**Roberto Bombardi, *MD***	Ospedale Nuovo, Ambulatorio malattie demielinizzanti del SNC, Unita' Operativa Complessa di Neurologia, Conegliano, Italia
**Alberto Gajofatto, *MD***	Policlinico G.B. Rossi, Clinica Neurologica, Dipartimento di Neuroscienze, Biomedicina e Movimento, Verona, Italia
**Paolo Giannetti, *MD***	Ospedale Belcolle, Centro Sclerosi Multipla, Viterbo, Italia
**Claudio Solaro, *MD***	Casa Di Cura Mons. Luigi Novarese, Dip. Di Riabilitazione, Moncrivello VC, Italia
